# Training Enhances Immune Cells Mitochondrial Biosynthesis, Fission, Fusion, and Their Antioxidant Capabilities Synergistically with Dietary Docosahexaenoic Supplementation

**DOI:** 10.1155/2016/8950384

**Published:** 2016-09-06

**Authors:** Carla Busquets-Cortés, Xavier Capó, Miquel Martorell, Josep A. Tur, Antoni Sureda, Antoni Pons

**Affiliations:** ^1^Research Group on Community Nutrition and Oxidative Stress, Science Laboratory of Physical Activity, Department of Fundamental Biology and Health Sciences, University of Balearic Islands, 07122 Palma de Mallorca, Spain; ^2^Departamento de Nutrición y Dietética, Facultad de Farmacia, Universidad de Concepción, 4070386 Concepción, Chile; ^3^CIBER: CB12/03/30038 Fisiopatología de la Obesidad la Nutrición, CIBEROBN, Instituto de Salud Carlos III (ISCIII), University of Balearic Islands, 07122 Palma de Mallorca, Spain

## Abstract

Exercise training induces adaptations in mitochondrial metabolism, dynamics, and oxidative protection. Omega-3 fatty acids change membrane lipid composition and modulate mitochondrial function. The aim was to investigate the effect of 8-week training and docosahexaenoic acid (DHA) supplementation (1.14 g/day) on the mitochondria dynamics and antioxidant status in peripheral blood mononuclear cells (PBMCs) from sportsmen. Subjects were assigned to an intervention (*N* = 9) or placebo groups (*N* = 7) in a randomized double-blind trial. Nutritional intervention significantly increased the DHA content in erythrocyte membranes from the experimental group. No significant differences were reported in terms of circulating PBMCs, Mn-superoxide dismutase protein levels, and their capability to produce reactive oxygen species. The proteins related to mitochondrial dynamics were, in general, increased after an 8-week training and this increase was enhanced by DHA supplementation. The content in mitofusins Mtf-1 and Mtf-2, optic atrophy protein-1 (Opa-1), and mitochondrial transcription factor A (Tfam) were significantly higher in the DHA-supplemented group after intervention. Cytochrome c oxidase (COX-IV) activity and uncoupling proteins UCP-2 and UCP-3 protein levels were increased after training, with higher UCP-3 levels in the supplemented group. In conclusion, training induced mitochondrial adaptations which may contribute to improved mitochondrial function. This mitochondrial response was modulated by DHA supplementation.

## 1. Introduction

Assembly of new mitochondria in eukaryotic cells occurs in response to the energy demands of the cell in a process called mitochondrial biogenesis. In addition, this event permits the equal distribution of mitochondria among the two cells formed when cell division occurs. Mitochondria form a dynamic tubular reticulum within eukaryotic cells and the generation of their components requires the lipids and proteins synthesis by nuclear genome and the mitochondrial DNA expansion from the preexisting mitochondrial reticulum [1–3]. Due to its reticular properties and high plasticity, mitochondria can rapidly change its size, shape, and distribution by constantly alternating fusion and fission processes [3]. The merging of discrete mitochondria (fusion) to the reticulum leads to the configuration of a continuous mitochondrial network that allows the preservation of mitochondrial content and structural homeostasis. The fragmentation in the reticulum (fission) permits the separation of impaired mitochondria from the healthy network, improving and maintaining mitochondrial quality [4]. Fusion and fission, strictly regulated and permanently balanced, allow the cell to reorganize its mitochondrial network and maintain the equilibrium needed to keep mitochondrial morphology and function but can shift the balance in order to cope with the changing physiological demands [5]. In mammals fission, it is controlled by dynamin-like GTPases such as dynamin-related protein 1 (DRP1) and fission protein 1 (FIS-1) [1]. Concretely, fission followed by selective fusion segregates dysfunctional mitochondria and permits their removal by mitophagy and also participates in cell apoptosis by releasing cytochrome c [6]. On the contrary, fusion processes are controlled by the membrane-bound GTPases mitofusin 1 (Mfn-1), mitofusin 2 (Mfn-2), and optic atrophy protein 1 (Opa-1), which are essential for combination of the outer and inner mitochondrial membrane [7]. The process of fusion generates networks with continuous membranes and matrix lumens. As a result, not only metabolites, solutes, and proteins can be exchanged among constituent mitochondria but they also share electrochemical gradient, being capable of dissipate metabolic energy by transmission of membrane potential [8]. These phenomena of fusion and fission show that mitochondrial dynamics plays a central role in controlling cell viability and any error resulting from malfunction can cause cell problems that may lead to serious diseases in mammals [9].

Dietary fatty acids have been described to exert diverse effects on mitochondrial function and dynamic behaviour. While saturated fatty acids are commonly associated to cardiovascular diseases and their excessive consumption is not recommended, omega 3 polyunsaturated fatty acids have been reported to have beneficial effects on mitochondria by improving mitochondrial function and promoting mitochondrial fusion in both* in vivo* and* in vitro* experiments [10]. Docosahexaenoic acid (DHA) is an essential omega-3 polyunsaturated fatty acid (PUFA) mainly found in marine food. Together with eicosapentaenoic acid (EPA), DHA is recognized as a protective molecule against inflammation and oxidative stress and it also promotes the gene expression of key enzymes that introduce fatty acids into the mitochondria and their use as energetic fuel in the respiratory chain [11]. However, the high unsaturation index of long-chain omega-3 PUFA, especially DHA, may make them prone to peroxidation, which may be associated with oxidative processes [12, 13]. Thus, prooxidant and antioxidant properties of omega 3 fatty acids and their physiological effects are still controversial.

Mitochondria are known for initiating signal transduction cascades to the nucleus that coordinate transcriptional responses after physiological perturbations. In skeletal muscle, contraction processes cause an increase in reactive oxygen species (ROS) production and a transient situation of oxidative stress. This transitory oxidative stress induced by exercise has an important role in muscle signalling through release of ROS, Ca^2+^, metabolites, and myokine levels. Mitochondrial and nonmitochondrial adaptations that restore and maintain the cellular homeostasis are responsible for beneficial outcomes of physical activity, when practised moderately [13]. Skeletal muscle adaptive response to training is translated into increased endurance, enhanced vascular function (angiogenesis), and oxidative myofiber transformation, among others. Concerning mitochondria, training confers healthy benefits due to coordinated improvements in quality (structure and function) and quantity (content) through structural joining (fusion) and separation (fission) and synthesis-incorporation of proteins and mitochondrial DNA in the existing reticulum (biogenesis) [4]. Besides, an improvement in cardiac and skeletal muscle mitochondrial respiratory capability and a proper regulation of mitochondrial life cycle span occur [14, 15]. Physical activity induces molecular adaptive responses mediated by oxidative stress and mitochondrial function including the NF-*κβ* pathway, which activates target genes related to antioxidant defenses like uncoupling proteins (UCPs) and the mitochondrial biogenesis mediated by peroxisome proliferator activated receptor gamma (PGC-1*α*). However, this signalling cascade clearly evidenced in skeletal muscle is poorly studied in other cell types. We hypothesise that the oxidative stress induced by regular training can affect cell functionality and, thereby, can exert some effects on mitochondrial dynamics that have not been described in PBMCs.

Our aim was to investigate the effect of a long-term training and DHA supplementation on the antioxidant status and on the expression of mitochondrial biogenesis proteins in immune cells from professional football players. Obtaining muscle cells to investigate the mitochondrial adaptations to exercise is complex and it implies an invasive procedure with many ethical difficulties in healthy subjects. Accordingly, we selected peripheral blood mononuclear cells (PBMCs) as a cellular model for studying the mitochondria dynamics in a quicker and easier way than muscle biopsies and to validate their usefulness in evidencing changes associated with exercise.

## 2. Materials and Methods

### 2.1. Subjects Characteristics and Nutritional Intervention

At the beginning of the competition season, 23 male professional and federated football players (Real Mallorca B) were recruited to take part in the study. Unfortunately, from all subjects selected, only 16 reached the end of study because 6 of them left the football team during the experimental time and joined the professional team, and one player broke the anterior cruciate ligament of the knee. Subjects were randomly classified into two groups: placebo, composed of 7 subjects, and experimental, with 9 subjects. Participants in the study were healthy and nonsmokers. The anthropometric and physical performance characteristics of participating football players were as follows: 18.9 ± 0.5 years old (placebo group) and 20.4 ± 0.5 years old (experimental group), 76.0 ± 1.5 kg of weight (placebo group) and 76.4 ± 3.5 kg (experimental group), and 181 ± 3 cm of height (placebo group) and 180 ± 3 cm (experimental group). The waist circumference was 78.0 ± 0.7 and 78.5 ± 1.1 cm, the hip circumference was 99.0 ± 1.6 and 96.6 ± 1.4 cm, and the waist–hip ratio (WHR) was 0.787 ± 0.018 and 0.814 ± 0.012, in placebo and experimental groups, respectively. The value of systolic blood pressure was 115 ± 6 and 120 ± 4 in placebo group and 56.7 ± 5.9 and 66.7 ± 3.5 mmHg in experimental groups. The body mass index (BMI) was 23.1 ± 0.4 and 23.5 ± 0.5 kg/m^2^, and the football players had 91.5 ± 0.3 and 92.8 ± 0.3% fat-free mass, in placebo and experimental groups, respectively. The VO_2max_ in placebo group was 61.2 ± 1.6 and 62.0 ± 0.9 mL/kg min in experimental group, respectively. Finally, the intense physical activity time was 97.5 ± 58.3 and 52.0 ± 13.7 and 68.6 ± 17.1 and 63.2 ± 14.6 min/day of moderate physical activity time, in placebo and experimental group, respectively. There were no differences in these parameters neither between the placebo and experimental groups of football players nor between initial and final nutritional intervention. Following a randomized double-blind trial, participants in the study ingested daily 1 litre of placebo or experimental drink for 5 days per week (excluding the match day and the day of rest), for a period of 8 weeks of training. The beverages were consumed before starting the physical activity session. The training performed during the experimental time consisted of six physical activity sessions per week and 10 official matches. The exercise consisted of 2 h regular football training [15]. All the subjects were properly informed of the purpose and demands of the study before giving their written consent to participate. The study protocol was in accordance with the Declaration of Helsinki for research on human subjects and was approved by the Ethical Committee of Clinical Investigation of the Autonomic Community of the Balearic Islands No. IB 994/08 PI (Palma de Mallorca, Balearic Islands, Spain). The project was registered at ClinicalTrial.gov (NCT02177383).

### 2.2. Drink Composition

The beverages were composed of 3.0% almond, 0.8% sucrose, water, lemon and cinnamon and *α*-tocopherol acetate (vitamin E), and 0.8% of different lipids depending on the kind of beverage (placebo or experimental). The lipid content of the placebo drink was 0.8% refined olive oil, and for the experimental drink was 0.6% olive oil and 0.2% DHA-S Market (Market Biosciences Corporation, Columbia, EEUU). The two almond drinks were manufactured by Liquats Vegetals S.A. (Girona, Spain), and the procedure for obtaining them consisted of bleaching almonds and crushing them in water. Then, the mixture was centrifuged to eliminate insoluble materials and cinnamon, lemon natural flavours, sucrose, and vitamin E were added. Finally, olive oil was added to the placebo drink and olive oil plus DHA-S to the experimental one, but there were no differences in taste. Finally, the beverage was sterilized and packed into apparently identical bottles. The concentration of vitamin E in both placebo and experimental drinks is equivalent to 0.4 mg/mL of *α*-tocopherol acetate.

### 2.3. Experimental Procedure

A total of two venous blood samples were obtained from each subject over the experimental time in basal conditions after overnight fasting. One sample was extracted before starting the nutritional intervention and the other one at the end of the nutritional intervention. Both samples were obtained from the antecubital vein of subjects in suitable vacutainers with EDTA as anticoagulant and were immediately used to purify erythrocytes and PBMCs. Cell counts were determined in an automatic flow cytometer analyzer Technicon H2 (Bayer, Leverkusen, Germany) VCS system.

The PBMCs fraction was purified using Ficoll-Paque PLUS reagent (GE Healthcare, Chalfont St Giles, UK) [16, 17] Briefly, blood was carefully introduced on Ficoll in a proportion of 1 : 5 : 1 and was then centrifuged at 900 ×g, at 4°C for 30 min. The PBMCs layer was carefully removed while plasma and Ficoll phases were discarded. The PBMCs slurry was then washed twice with PBS and centrifuged for 10 min at 1,000 ×g, 4°C. This process was performed in duplicate: one of the samples was destined to obtaining RNA and the other one was preserved in RIPA lysis and extraction buffer (250 mM Tris/HCl, pH 8.0, 4.4% NaCl, 5% IGEPAL®, 2.5% deoxycholic acid, 0.5% sodium dodecylsulfate (SDS)) for Western blot analysis. Cell lysates were stored at −80°C until biochemical analyses.

Erythrocytes were purified by centrifugation at 900 ×g at 4°C for 30 min. The erythrocyte phase at the bottom was washed with PBS, centrifuged as above, and finally erythrocytes were reconstituted with distilled water. Fatty acid extraction of erythrocyte samples was performed by a modification of the Folch extraction procedure [17, 18]. The method for individual fatty acid determination in erythrocytes was previously described [19].

### 2.4. Hydrogen Peroxide (H_2_O_2_) Production

H_2_O_2_ production by PBMCs was measured before and after stimulation with phorbol myristate acetate (PMA) using 2,7-dichlorofluorescin-diacetate (DCFH-DA) as indicator. A stock solution of DCFH-DA (1 mg/mL) in ethanol and PMA (1 mg/mL) in DMSO was prepared and stored at 20°C until analysis. DCFH-DA (30 *μ*g/mL) in PBS was added to a 96-well microplate containing 50 *μ*L PBMCs suspension. PMA (10 ng/mL) prepared in HBSS or HBSS alone was added to the wells, and the fluorescence (Ex, 480 nm; Em 530 nm) was recorded at 37°C for 1 h in FL 9800 Microplate Fluorescence Reader (Bio-Tek Instruments, Inc.).

### 2.5. SDS-Polyacrylamide Gel Electrophoresis and Western Blot Analysis

Antioxidant enzyme and mitochondrial protein levels were determined by Western blot analysis. PBMCs lysed with RIPA buffer were heated for 5 min at 100°C. 30-microgram protein aliquots were loaded in each lane and separated by size using SDS polyacrylamide gel (12% acrylamide) and electrotransferred onto a nitrocellulose membrane. The membrane were blocked (5% non-fat powdered milk in PBS, pH 7,5, containing 0,1% Tween 20) and incubated with the corresponding primary monoclonal antibody. Antibodies for PGC-1*α* (1 : 1000), Mfn-1 (1 : 500), Mfn-2 (1 : 200), OPA-1 (1 : 500), metalloendopeptidase 1 (OMA1) (1 : 200), FIS1 (1 : 200), actin (1 : 200), and UCP-2 (1 : 500) were supplied by Santa Cruz Biotechnology (Santa Cruz, Ca, USA); anti-nuclear respiratory factor 1 (NRF1) (1 : 1000) and anti-mitochondrial transcription factor A (Tfam) (1 : 1000) antibodies were obtained from Cell Signalling (Danvers, MA, USA); antibodies against UCP-3 (1 : 500), Mn-super oxide dismutase (Mn-SOD) (1 : 1000) and cytochrome c oxidase subunit 4 (COX-IV) (1 : 1000) were from Millipore (Billerica, MA, USA). Blots were then incubated with a secondary peroxidase-conjugated antibody (1 : 5000). Development of immunoblots was performed using and enhanced chemiluminescence kit (Immun-Star® Western C® Kit reagent, Bio-Rad Laboratories). Protein bands were visualized using the image analysis program Quantity One (Bio-Rad). Precision Plus Protein™ Kaleidoscope™ (Bio-Rad) was used as a molecular weight marker. The band density of each protein was quantified in relation to the loading control (actin), used as a housekeeping.

### 2.6. Malondialdehyde Assay

Malondialdehyde (MDA) in PBMCs was analyzed by colorimetric assay for MDA determination, based on the reaction of MDA with a chromogenic reagent that produces a stable chromophore with maximal absorbance at 586 nm. Succinctly, samples or standards were derivatizated using 1-methyl-2-phenylindole (10.3 mM) in acetonitrile : methanol (3 : 1). Proteins were precipitated with HCl 12 N and the samples were incubated for 1 h at 45°C. Absorbance was then measured at 586 nm.

### 2.7. Protein Carbonyl Determination

Protein carbonyl derivatives were determined through immunological methods using the OxiSelectTM Protein Carbonyl Immunoblot Kit (Cell Biolabs, INC) by following the manufacturer's instructions. Protein concentrations of an aliquot of cells lysed with distilled water were calculated by the Bradford method [20] using the Bio-Rad protein assay reagent (Bio-Rad, Munich, Germany) and 10 *μ*g of protein was transferred onto a nitrocellulose membrane by the dot blot method. Briefly, samples were placed in a vacuum plate and absorbed. A derivatization protocol was followed, using methanol 50%, HCl 12 N, and 2,4-dinitrophenylhydrazine as reagents. Then, the membrane was incubated with the primary antibody, specific to DNP moiety proteins (1 : 4,000). After this, an incubation with a secondary horseradish peroxidase-conjugated antibody (goat anti-rabbit IgG) (1 : 10,000) was performed. The membrane was finally treated with luminol, which is transformed to a light-emitting form at 428 nm through the antigen/primary antibody/secondary antibody/peroxidase complex. The resulting light was visualized by short exposure to a Chemidoc XRS densitometer imaging system (Bio-Rad Laboratories) and bands quantification was performed by using Quantity One-1D analysis software (Bio-Rad Laboratories).

### 2.8. Statistical Analysis

Statistical analysis was carried out using the Statistical Package for Social Sciences (SPSS v.19.0 for Windows). Results are expressed as mean ± SEM, and *p* < 0.05 was considered statistically significant. A Kolmogorov–Smirnov test was applied to assess the normal distribution of the data. The statistical significance of the data was assessed by two-way analysis of variance (ANOVA). The statistical factors analyzed were beverage supplementation (*S*) and the training period (*T*). The sets of data in which there was a significant interaction between the factors analyzed were tested by one-way ANOVA. When significant effects of one factor were found, a Student's *t* test for paired data was used to determine the differences between the groups involved.

## 3. Results

Diet supplementation with DHA-enriched beverage for 8 weeks changed lipid composition of erythrocytes membranes. No differences were observed between placebo (DHA concentration was 29.0 ± 1.3 nmol/10^9^ erythrocytes) and experimental (DHA concentration was 34.0 ± 3.6 nmol/10^9^ erythrocytes) groups at the beginning of the study. The erythrocyte from the experimental group increased their content in DHA (43.0 ± 3.7 nmol/10^9^ erythrocytes) after 8 weeks of nutritional intervention whereas no effects were observed in placebo group (33.6 ± 3.2 nmol/10^9^ erythrocytes). These results imply an increase of 26% in the DHA content with respect to the initial values in erythrocytes from the experimental group.

**Figure 1:**
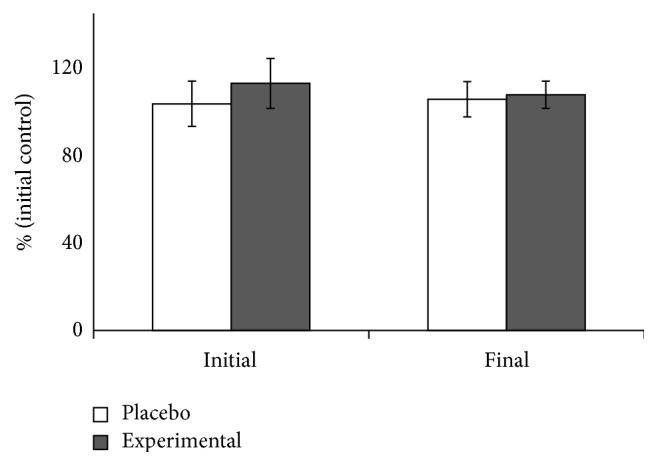
Effects of training and DHA supplementation on ROS production by PBMCs after PMA stimuli. Statistical analysis: two-way ANOVA, *p* < 0.05. No significant differences were reported.

Total PBMC counts and the % of lymphocytes and monocytes were reported in Table 1. No significant differences between placebo and experimental groups or the training period are observed in any cellular parameter. No effects of 8 weeks training or DHA supplementation were evidenced on the ROS production capabilities of PBMCs in response to PMA stimulation (Figure 1). The MDA levels in PBMCs significantly decreased in placebo and in experimental groups after the 8-week training season, while carbonyl index significantly increased in both groups (Table 3).

**Figure 2:**
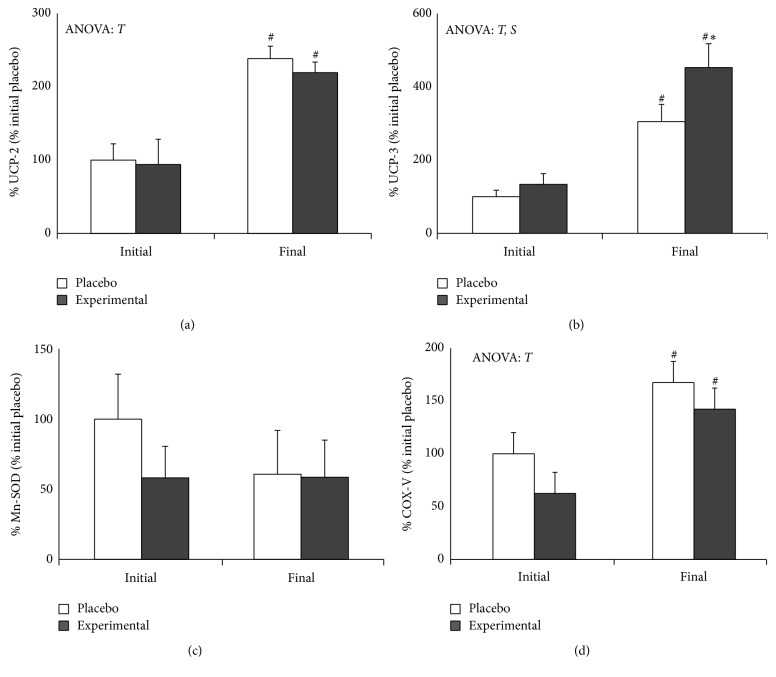
Effects of training and DHA supplementation on mitochondrial protein levels of PBMCs. (a) uncoupling protein- (UCP-) 2, (b) UCP-3, (c) Mn-superoxide dismutase (Mn-SOD), and (d) cytochrome c oxidase (COXIV). Statistical analysis: two-way ANOVA, *p* < 0.05. *T*, significant effect of training; *S*, significant effect of DHA supplementation. One-way ANOVA, *p* < 0.05. (*∗*) Significant differences between placebo and experimental groups; (#) significant differences between initial and final training period.

Figure 2 shows the effect of 8 weeks of training and DHA supplementation on the mitochondrial antioxidant and cytochrome c oxidase protein levels. No effects of training or DHA diet supplementation were observed on Mn-SOD (Figure 2(c)) protein levels; however, training significantly increases protein levels of UCP-2 (Figure 2(a)) and COX-IV (Figure 2(d)) in both placebo and experimental groups. These results are similar to those obtained for UCP-3 (Figure 2(b)) protein levels where a significant increase due to training is observed, but in this case the increase was enhanced by DHA diet supplementation.

**Figure 3:**
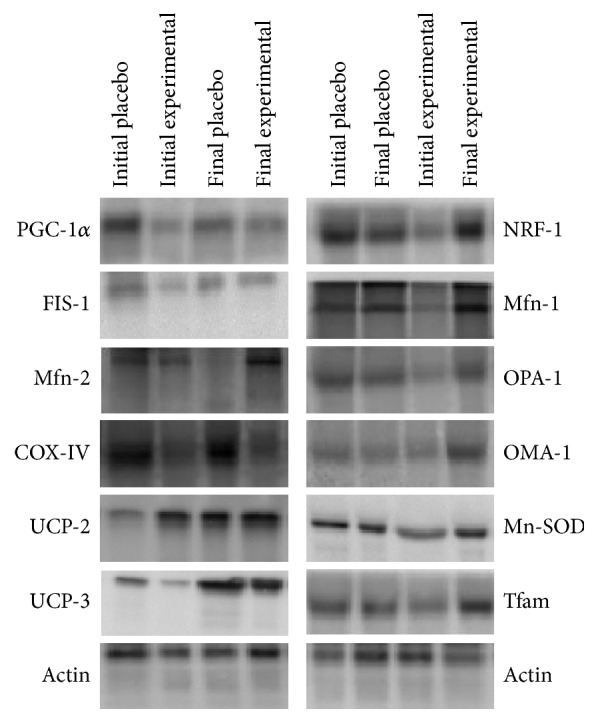
Representative picture of the bands obtained by immunoblotting.

The effects of training and DHA diet supplementation on mitochondrial dynamics are shown in Table 2 and Figure 3. PGC1*α*, NRF-1, and OPA-1 protein levels were influenced by the 8-week training period. OPA-1 protein levels significantly increased after training in both placebo and experimental groups, whereas in PGC1*α* and NRF-1 protein levels the increase was only significant in experimental group. Additionally, Tfam and OMA-1 protein levels were affected by training but also were influenced by DHA diet supplementation. OMA-1 and Tfam protein levels increased after the training in both groups but this increase was significantly higher in experimental group. On the other hand, Mfn-1 and Mfn-2 were affected by training and DHA diet supplementation and by interaction of both factors, reporting a significant increase only in the experimental group after the training period. No effects of training season or DHA supplementation were observed on FIS-1 protein levels.

## 4. Discussion

The main feature of this study was to evidence the influence of exercise training and dietary supplementation with DHA on the peripheral blood mononuclear cells mitochondrial dynamics and antioxidant function. Regular training induces a greater ROS production in cells, but it also triggers expression of antioxidants enzymes and protection against PBMCs' lipid peroxidation [21]. However, we detected an increase in the levels of protein carbonyl derivates. Oxidative alterations in proteins include the generation of protein carbonyls as a modification of single amino acids [22]. The MDA-amino group reaction can also promote the introduction of carbonyls groups into the protein [23]. We suggest that the lower levels of MDA in PBMCs at the end of the 8-week training period could be a consequence of the MDA reaction with proteins, fact that would increase the carbonyl index.

Eight weeks of regular training enhanced the antioxidant mitochondria capabilities to decrease mitochondrial ROS production through increasing UCP-2 and UCP-3 protein levels; it also improves processes of mitochondrial biosynthesis, fission and fusion that were favoured by the DHA dietary supplementation. Some of these training effects have also been previously described in skeletal muscle as a consequence of the contraction process [24, 25]. Thus, we can translate this exercise-induced muscular response into changes in peripheral blood mononuclear cells, establishing a first approach between immune system cells mitochondrial dynamics and skeletal muscle cells model. Since no differences were reported in PBMC counts and the percentage of lymphocytes and monocytes in both placebo and experimental groups, changes in mitochondria markers may be attributed to training and/or DHA incorporation in PBMC membrane and not to changes in the lymphocyte/monocyte ration.

Both beverages (placebo and experimental) had the same nutritional basis. Concretely, they contain *α*-tocopherol and phenolic compounds from almond, such as catechin, quercetin, and kaempferol, which have been demonstrated to exert potent free radical scavenging activity and are considered dietary beneficial antioxidants [26–29]. These bioactive ingredients can modulate oxidative stress in cells, but the experiment was designed to study the effect of DHA on PBMCs and not to study the effect of the almond and olive oil based beverage. Consequently, placebo and experimental beverages only differed in the content of DHA, while the rest of the compounds in the beverages were the same. According to our experimental design and taking into account that physical activity parameters were comparable in all football players, observed changes can be attributed to DHA action, but we are not able to discard other bioactive compounds' contributions to counteract oxidative stress when training effect was evidenced in PBMCs. Further studies including a control group supplemented with equally energetic and mineral content beverage but deprived of other bioactive compounds and DHA would be necessary to differentiate the effect of training and the effect of the dietary almond and olive oil based beverage supplementation on PBMCs antioxidant status. There is a lack of studies performed about effects of DHA on mitochondrial dynamics, so any change evidenced with our experimental design could be attributed to DHA action and should be taken into consideration.

Erythrocytes cell membranes are usually used as biomarkers of medium-term fatty acids intake as they reflect the proportion accumulated over the lifespan of red blood cells [30]. In our case, a DHA-enriched diet for 8 weeks induced a change in lipid composition of erythrocytes membranes of football players; those who ingested the experimental drink increased their DHA content compared to those who ingested the placebo drink. This indicates that the participants followed the prescribed beverage intake during the trial and reinforces the idea that DHA can incorporate into immune cell membranes and may exert some effects on peripheral blood mononuclear cells function, even on its mitochondrial dynamics. In fact, 8 weeks of dietary omega-3 supplementation attenuates proinflamatory cytokine production after a bout of acute exercise [18] and enhances antioxidant defenses in professional athletes under resting conditions and after acute exercise [15].

The capability to modulate mitochondrial function and number is a prominent adaptive response in all eukaryotic cells. In mammals there exist various factors that trigger this mitochondrial reaction, including hormone levels, aging, hypoxia, and local environmental stressors like temperature, physical exercise, and type of food ingested [31]. Concretely, dietary fatty acids have been suggested to exert some effects on mitochondrial dynamics. Beneficial effects of omega 3 polyunsaturated fatty acids have been reported, especially those derived from fish oil rich in EPA and DHA. These PUFAs improve mitochondrial function, promote mitochondrial fusion, and reduce ROS production in rat hepatic mitochondria [32]. In addition, it was described that 1 hour of incubation with omega 3 PUFA upregulates Mfn-2 expression and increases ATP levels in an* in vitro* steatotic hepatocyte model [33].

On the other hand, exercise training implies a reiterated exposure to an acute increase in metabolic, thermoregulatory, hypoxic, oxidative and mechanical stress. Oxygen overconsumption during acute and intense exercise increases ROS production [34] that can overwhelm the antioxidant defenses and lead the cell to an oxidative stress status [27, 28]. However, regular physical exercise originates adaptations in antioxidant defenses in muscle and immune cells and improves exercise performance [29, 30].

Mechanical stress-induced signals, such as p38 MAPK, which gets activated by the elevation of cytosolic Ca^2+^ during muscle contraction, have the potential to stimulate and regulate the activity and expression of exercise-sensitive transcription factors like PGC-1*α* [35]. Our obtained data are in accordance with these effects associated to exercise evidencing an increase in PGC-1*α*, which, in turn, induces mitochondrial biogenesis (fusion) by orchestrating transcription of nuclear genome (through interaction with NRF1) and mitochondrial genome (via Tfam gene transcription). These molecules provide a link between physiological stimuli and transcription of nuclear gene that induce compensatory physiological adaptations that enhance tolerance thresholds to subsequent sublethal doses of stressors [13]. Another PGC-1*α* target is COX-IV, the terminal oxidase in the mitochondrial electron transport chain and a mitochondrial amount indicator [36]. An increased COX-IV protein level expression in our PBMCs could be due to an augmented total mitochondrial number; thus, mitochondrial specific proteins such as mitofusins undergo a raise in their protein expression. This reinforces the idea that exercise promotes biogenesis processes. Studies performed in skeletal muscle associate increases in COX-IV protein levels with training resulting in an improvement in the capacity of mitochondria to produce ATP [37]. Mitofusins and OPA-1 have been characterized as the leading factors for mitochondrial fusion of the outer and inner mitochondrial membrane, respectively [38]. Exercise promotes this process, as it was demonstrated by significant increases in Mtf1/2 and OPA-1 protein levels after training period in both placebo and experimental groups. In addition to training, mitofusins were also influenced by DHA diet supplementation and by interaction of both factors. Fusion allows compensation of damage in mitochondria by sharing components and helps to maintain energy output to face stress situations [2]. Similar results were found in skeletal muscle of rats. Those fed with a high fat diet rich in fish oil diet (HFO) diminished fission process and augmented fusion processes compared to those fed with a high-lard diet (HL). Indeed, skeletal muscle sections from HFO fed rats revealed a greater number of immunoreactive fibers for Mfn2 and Opa1 protein, while sections from HL fed rats showed a weaker immunostaining for Drp1 and Fis1 [11, 24].

Overexpression of FIS-1 has been reported to induce mitochondrial fragmentation and apoptosis in HeLa cells [39] suggesting the involvement of mitochondrial fission in apoptosis. Even so, no effects of training season or DHA supplementation were observed on FIS-1 protein levels in our experiment. This may indicate that the addition of new mitochondria to the healthy network (fusion) outweighs the removal of the impaired ones (fission) under exercise conditions. Notwithstanding low levels of damage might be compensated by complementation through mitochondrial fusion; badly injured mitochondria can produce excessive amounts of ROS, by consuming ATP through reversal of ATP synthase. Therefore, rejoining of badly affected mitochondria will infect the healthy mitochondrial reticulum if they are not eliminated. OMA-1 is a metalloprotease involved in the quality system in the inner membrane of mitochondria. Stressing conditions like low levels of ATP or severely impaired mitochondrial activities provoke a loss of mitochondrial membrane potential that causes cleavage and inactivation of OPA-1 mediated by OMA-1, leading to a negative regulation of mitochondrial fusion. Then, the outer membranes of most seriously damaged mitochondria can still fuse but the inner membrane–bound matrix compartments fusion is prevented [1, 3, 40]. OMA-1 protein levels in our PBMCs increased after the training period in placebo and supplemented group, with this increase being significantly higher in the experimental one.

ROS produced during exercise causes a transient oxidative stress status that triggers the antioxidant regulatory mechanisms in immune system cells [41]. These adaptive responses include the NF-*κβ* pathway, which activates target genes related to antioxidant defenses such as the mitochondrial uncoupling proteins and antioxidant enzymes like Mn-SOD [42]. UCP-2 and UCP-3 are located in the mitochondrial inner membrane and catalyze a proton leak that uncouples oxidative phosphorylation and dissipates electrochemical gradient across the membrane. This may lead to a reduced ROS production [43]. In the present study regular training significantly increased the UCP-2 and UCP-3 protein levels in PBMCs bringing to light a possible augmented number of UCP-2/3 per mitochondrion in accordance with studies carried out in skeletal muscle [44] and in PBMCs [45]. The increment in UCP-3 was enhanced by DHA diet supplementation which is consistent with other studies carried out with mice cells that show that omega-3 may increase the expression of UCP-3 [46]. This may evidence a synergistic effect of DHA diet supplementation and training on PBMCs antioxidant capabilities. On the contrary, no changes were reported on the antioxidant enzyme Mn-SOD protein levels, suggesting that the increased levels of UCPs could be enough to reduce the exercise induced ROS overproduction. Moreover, subjects from the study were professional athletes and probably their basal antioxidant status allows them to protect against oxidative stress. In addition, the higher UCPs content that is reported to lead to a reduced ROS production could be also responsible of the lack of differences in ROS production between the beginning and the end of the training period. It is important to note that the levels of ROS are key to determine their physiological effect. Low/moderate ROS production rates are associated with their role as molecular signalling inducing adaptive responses, while higher levels are harmful and associated with processes of senescence and apoptosis. Consequently, avoiding excessive production of ROS is essential to ensure cell functionality and to potentiate the adaptive responses to exercise.

In conclusion, the present results evidenced that 8 weeks of regular training induces mitochondrial adaptations in terms of fusion and fission processes and in antioxidant defenses in PBMCs to meet the demands arising from exercise and this response is potentiated by DHA diet supplementation. Thus, our results could be an opening approximation to define a link between mitochondrial events in immune cells and skeletal muscle cells and to reveal that training improves gradually mitochondrial quantity (mitochondria biogenesis) and quality (balance between biogenesis, dynamics and mitophagy). Further work is needed to identify the molecular mechanisms in which omega 3 fatty acids perform their biological activities on mitochondria.

## Figures and Tables

**Table 1:** Effects of training and DHA supplementation on PBMC counts.

		Initial	Final	ANOVA		
				*S*	*T*	*S* × *T*
PBMCs (10^3^ cells/*μ*L)	Placebo	2.47 ± 0.6	2.98 ± 0.4			
	Experimental	2.92 ± 0.2	3.61 ± 3.6			

Lymphocytes (%)	Placebo	79.9 ± 6.6	82.9 ± 4.3			
	Experimental	85.1 ± 2.9	85.3 ± 3.6			

Monocytes (%)	Placebo	20.1 ± 5.5	17.0 ± 3.8			
	Experimental	14.9 ± 3.9	14.7 ± 3.3			

Statistical analysis: two-way ANOVA, *p* < 0.05. (*T*) significant effect of time of training, (*S*) significant effect of supplementation, (*S* × *T*) significant interaction between both factors. No significant differences were reported.

**Table 2:** Effects of training and DHA supplementation on mitochondrial dynamics protein levels of PBMCs.

		Initial	Final	ANOVA		
				*S*	*T*	*S* × *T*
PGC-1*α* (%)	Placebo	100 ± 15	152 ± 22		X	
	Experimental	65.3 ± 5.9	152 ± 29^#^			

NRF-1 (%)	Placebo	100 ± 19	148 ± 9		X	
	Experimental	59.5 ± 14.7	158 ± 23^#^			

Tfam (%)	Placebo	100 ± 14^a^	154 ± 24^b^		X	X
	Experimental	78.8 ± 23.7^a^	231 ± 15^c^			

Mfn-1 (%)	Placebo	100 ± 22^a^	129 ± 15^a^	X	X	X
	Experimental	92.6 ± 17^a^	250 ± 26^b^			

Mfn-2 (%)	Placebo	100 ± 19^a^	98.9 ± 11.9^a^	X	X	X
	Experimental	108 ± 33^a^	203 ± 29^b^			

OPA-1 (%)	Placebo	100 ± 23	217 ± 84^#^		X	
	Experimental	134 ± 19	218 ± 25^#^			

OMA-1 (%)	Placebo	100 ± 11^a^	163 ± 25^b^		X	X
	Experimental	64.8 ± 13.7^a^	219 ± 17^c^			

FIS-1 (%)	Placebo	100 ± 15	88.0 ± 27.4			
	Experimental	115 ± 16	125 ± 16			

Statistical analysis: two-way ANOVA, *p* < 0.05. (*T*) significant effect of time of training, (*S*) significant effect of supplementation, (*S* × *T*) significant interaction between both factors. One-way ANOVA, *p* < 0.05. (*∗*) significant differences between placebo and experimental, (#) significant differences between initial and final training period. When interaction exists between different groups, distinct letters (a, b, and c) reveal significant differences with respect to all other groups.

**Table 3:** Oxidative damage in PBMCs.

		Initial	Final	ANOVA		
				*S*	*T*	*S* × *T*
Malondialdehyde (nmol/10^9^ cells)	Placebo	440 ± 70	160 ± 10^#^		X	
	Experimental	510 ± 70	330 ± 40^#^			

Protein Carbonyls (%)	Placebo	100 ± 30	838 ± 78^#^		X	
	Experimental	94 ± 22	792 ± 65^#^			

Statistical analysis: two-way ANOVA, *p* < 0.05. (*T*) significant effect of training period, (*S*) significant effect of supplementation, (*S* × *T*) significant interaction between both factors. One-way ANOVA, *p* < 0.05. #: significant differences between initial and final training period.
